# Reliability of ultrasonography to detect inflammatory lesions and structural damage in juvenile idiopathic arthritis

**DOI:** 10.1186/s12969-018-0275-4

**Published:** 2018-09-17

**Authors:** Lucio Ventura-Ríos, Enrique Faugier, Laura Barzola, L. B. De la Cruz-Becerra, Guadalupe Sánchez-Bringas, Andrés Rodríguez García, Rocío Maldonado, Johannes Roth, Cristina Hernández-Díaz

**Affiliations:** 1 Laboratorio de ultrasonido musculoesquelético y articular, Instituto Nacional de Rehabilitación, Luis Guillermo Ibarra Ibarra, Calzada México-Xochimilco 289, Arenal de Guadalupe, Tlalpan, 14389 Mexico city, Mexico; 2 Reumatología Pediátrica, Hospital Infantil de México, Mexico city, Mexico; 3 Reumatología, Hospital de Niños Dr. Ricardo Gutiérrez, Buenos Aires, Argentina; 4 Hospital Universitario “Dr. José E. González”, UANL, Monterrey, Nuevo León Mexico; 5 Embriology Department, Medicine School, Universidad Nacional Autónoma de Mexico, Mexico City, Mexico; 6 Division of Pediatric Dermatology & Rheumatology, Children’s Hospital of Eastern Ontario, Ottawa, Canada

**Keywords:** Reliability, Musculoskeletal ultrasound, Juvenile idiopathic arthritis

## Abstract

**Background:**

Musculoskeletal Ultrasonography (MSUS) is an important tool for the clinical assessment in Juvenile Idiopathic Arthritis (JIA). The objective of this study was to evaluate the reliability of MSUS to detect elementary lesions: synovitis, tenosynovitis, cartilage damage and bone erosions in the wrist and metacarpal (MCP) joints of patients with JIA.

**Methods:**

Thirty children in various subgroups of JIA according to ILAR criteria, were included in this cross-sectional study. Clinical data including painful, swollen and limited joints were recorded. Five rheumatologist ultrasonographers, blinded to the clinical evaluation, evaluated the presence of elementary lesions in the wrist and MCP 2 and 3 joints bilaterally. The synovitis was graded in B-Mode and Power Doppler (PD). In addition to descriptive statistics intra- and inter-observer reliability was calculated using Cohen’s kappa according to Landis and Koch.

**Results:**

US detected more synovitis than the clinical examination (62% vs 28%, 30% vs 23% and 22% vs 17% in the wrist, second and third MCP joints respectively). The intra-observer concordance for synovitis in all joints was excellent in B-Mode (k 0.84 .63–1.0 *p* = 0.001), except for MCP 2, where it was good (0.61, IC 95% .34–89, *p* = 0.001). For both modalities (PD, B-Mode) tenosynovitis, cartilage damage and bone erosions it was also excellent. Regarding synovitis grading the concordance was excellent for all grades (0.83–1.0, IC 95% 0.51.1.0, *p* = 0.001), except for grade 1 where it was good (0.61, IC 95% 0.43–.83, *p* = 0.001). Reliability inter-observer for grayscale synovitis (0.67–0.95, IC 95% 0.67–1.0, *p* = 0.001), tenosynovitis grayscale (0.89, IC 95% 0.78–0.99, p.001), damage cartilage (0.89, IC 95% 0.78–0.99, *p* = 0.001), PD (0.66, IC 95% 0.39–1.0, *p* = 0.001). The concordance for grading synovitis was excellent, but for grayscale grade 1 and 2 (.66, IC 95% .53–.74, *p* = 0.007) and PD grade 1 and 2 (0.63, IC 95% .58–.91, *p* = 004) was good.

**Conclusions:**

The intra- and inter-observer reliability of MSUS for inflammatory and structural lesions is good to excellent for the wrist and MCP in patients with JIA.

## Background

In recent years, musculoskeletal ultrasound has been recommended for the evaluation of treatment response and the detection of subclinical synovitis in rheumatoid arthritis [1]. MSUS may be equally important in children [2]. It has many advantages over other imaging techniques being easily accessible, fast, dynamic, not exposure to radiation and not requiring sedation [3]. It is being used increasingly for patients with juvenile idiopathic arthritis (JIA) to confirm suspected clinical findings, subclinical synovitis, define specific anatomic structures and guide interventional procedures like joint injections [4–6]. MSUS has been shown to be superior to the clinical exam in the detection of synovitis in JIA [7, 8]. Despite the increasing use in daily clinical practice, there is still a lack of data on the reliability of MSUS in the evaluation of joint inflammation and structural damage [9]. Magni-Manzoni S et al. evaluated 52 joints demonstrating excellent intra- and inter-observer reliability in the assessment of synovitis by B-Mode and Power Doppler (PD) [7]. Two other studies reported good and excellent intra- and inter-observer reliability specifically in MCP joints [8, 9]. However, these studies were done before pediatric-specific definitions of synovitis have been established.

The pediatric joint does display unique features on MSUS and the lack of a MSUS definition of synovitis in JIA may have contributed to the lack of data on the reliability of MSUS assessments. Recently, the OMERACT-US Pediatric subtask force has provided the basis for the standardized assessment by defining MSUS features of synovitis [10]. This group also proposed a synovitis scoring system and showed good reliability of it in four joints including the wrist and second MCP [11]. No pediatric specific MSUS definitions exist yet for tenosynovitis, cartilage damage and bone erosions but these lesions have been defined for adults [12, 13].

The objective of this study was to evaluate the reliability of MSUS to detect synovitis, tenosynovitis, bone erosions and cartilage damage in the wrist and MCP joints of patients with JIA.

## Methods

This research was conducted in compliance with the declaration of Helsinki and the study was approved by the research ethics committee at the National Institute of Rehabilitation under protocol number 52/16.

Thirty children were recruited for this study from the Pediatric rheumatology service at the Hospital Infantil de México, all diagnosed with JIA according to the ILAR criteria independently of clinical status [14]. One pediatric rheumatologist performed the clinical exam to detect painful, swollen and limited joints on 2 consecutive days (fifteen children every day). The MSUS was done in two rounds on each child by five rheumatologists with variable experience in pediatric MSUS who were blind to the clinical evaluation (fifteen children every day). After clinical examination, the child was evaluated by the ultrasonographers. The wrist (radio-carpal and mid-carpal joint recesses), 2nd and 3rd metacarpophalangeal (MCP) joints were evaluated bilaterally. Prior to the intra and interobserver exercise, the rheumatology sonographers completed a session to review definitions of synovitis, tenosynovitis, cartilage damage, and erosions. Before to the assessments, written informed consent was taken from all the study participants and their parents/guardians.

Clinical characteristics such as age, gender, time since disease diagnosis, painful and swollen joint count, number of joints with limited range of motion, erythrosedimentation rate (ESR) and C-reactive protein (CRP) were recorded.

### MSUS definition of pathology, scanning protocol and equipment

**Fig. 1:**
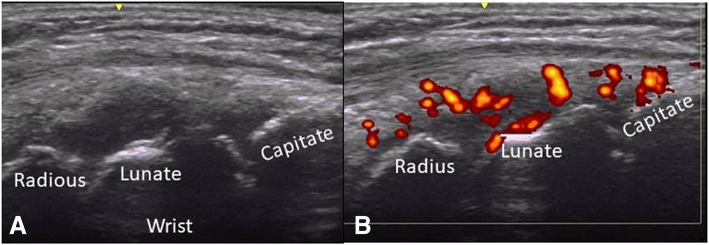
**a** Synovitis (grayscale) grade 2 in wrist. **b** Power Doppler grade 2. Both in longitudinal view

**Fig. 2:**
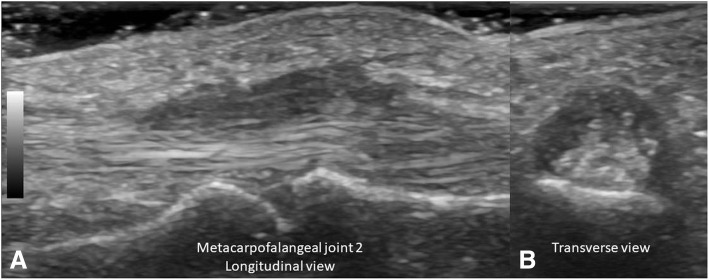
**a** and **b** Tenosynovitis of flexor tendon in longitudinal and transverse view, respectively

**Fig. 3:**
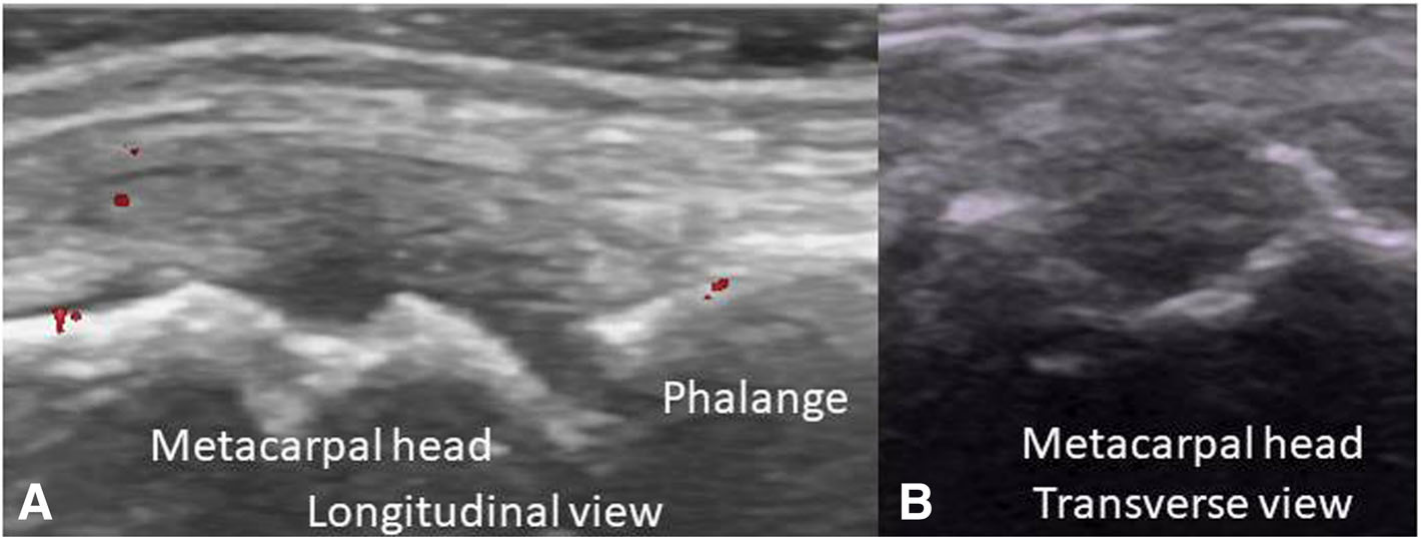
**a** and **b** Bone erosion of metacarpal head in longitudinal and transverse view

**Fig. 4:**
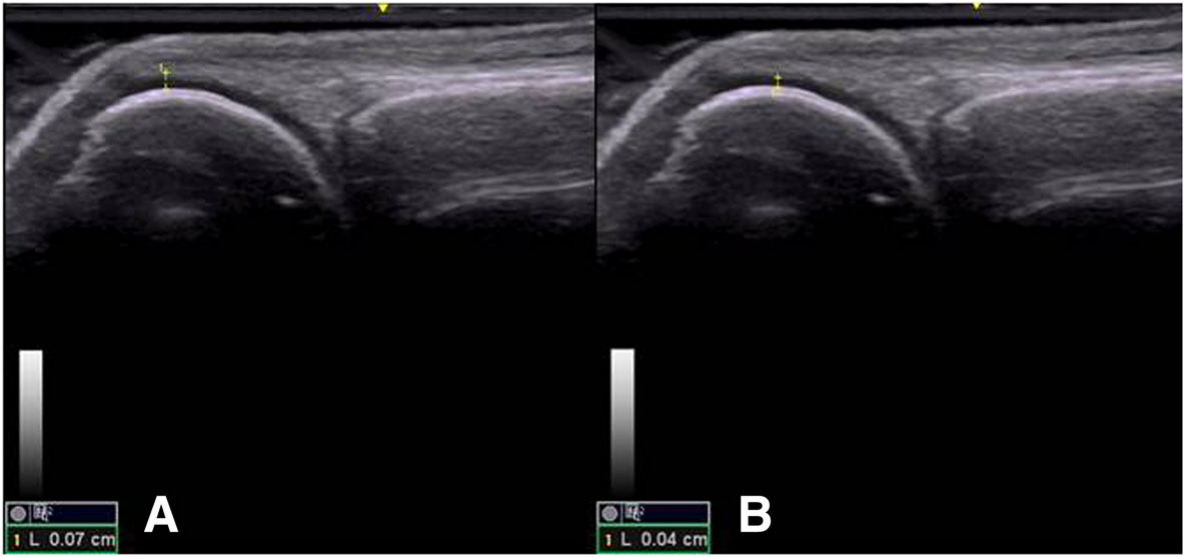
**a** Bone cartilage with a well-defined border. **b** Bone cartilage with irregular border and reduced thickness

According to the OMERACT pediatric MSUS definitions for synovitis, synovial effusion was defined as abnormal intra-articular fluid that is anechoic or hypoechoic and displaceable. Synovial hypertrophy as intra-articular and hypoechoic material that is non-displaceable. For pathologic Doppler signals, the term abnormal Doppler signals was used to differentiate to normal Doppler signal in normal tissue as suggested by OMERACT-US Pediatric subtask force [10]. The abnormal Doppler signals have to be shown within an area of synovial hypertrophy [11]. The primary goal of the study was a dichotomous assessment of synovitis being present or absent but in addition grading was done as well to demonstrate the degree of synovitis present in our patients. This was also done in light of the fact that there is currently no agreement on the significance of low grade Doppler signals. We used a scoring system developed by the OMERACT pediatric ultrasound task force to graduate B-mode findings as follows: Grade 1: synovial effusion and/or synovial hypertrophy that leads to a mild change of the joint recess appearance, Grade 2: synovial effusion and/or synovial hypertrophy that leads to a moderate change of the joint recess appearance (Fig. 1a) and Grade 3: synovial effusion and/or synovial hypertrophy that leads to a severe change of the joint recess appearance. For PD Grade 1 was defined as a detection of up to 3 single Doppler signals within the area synovial hypertrophy with or without normal physiological Doppler signals, Grade 2 as the detection of more than 3 Doppler signals but less than 30% of the area of synovial hypertrophy with or without normal physiological Doppler signals (Fig. 1b) and Grade 3 as the detection of Doppler signals within more than 30% of the area of synovial hypertrophy with or without normal physiological Doppler signals [10]. For tenosynovitis, the OMERACT definition for rheumatoid arthritis was used. It is defined as an abnormal, hypoechoic or anechoic (relative to tendon fibers) tendon sheath widening [12] (Fig. 2a, b). Similarly bone erosions were defined according to OMERACT as an intra-articular discontinuity of the bone surface visible in 2 perpendicular planes [12] (Fig. 3a, b). To evaluate cartilage thickness, the child were placed in supine position with both hands palm-side down on the examination table, then measurements of the cartilage thickness of the second and third MCP was obtained from a longitudinal dorsal scan with the MCP joints in a 90-degree flexion [15] (Fig. 4a, b). In this position, the delineation of cartilage of the epiphyses of the metacarpal head and proximal phalange is better [9]. Tenosynovitis, cartilage damage and bone erosions were evaluated dichotomously as absent/present.

The views to assess the wrist and MCP on MSUS were chosen according to the recommendations for standardized scanning by the OMERACT pediatric ultrasound group [16]. With the palm facing downwards, the wrist was assessed in neutral position with the transducer placed longitudinally in sagittal midline of the wrist, the proximal end of transducer positioned just distal to the radius diaphysis. The 2nd MCP joint was assessed with the palm facing downwards, laterally, or upwards and the finger was positioned flat in neutral position with the transducer placed longitudinally to evaluate the dorsal, lateral and volar aspect of the MCP joint. For the 3rd MCP joint, only the dorsal and volar aspects were evaluated [16].

Two GE NEXTGEN LOGIQ e R6 ultrasound machines with L8-18i-RS wide band linear were used for the assessments. Low flow settings were used for Power Doppler with a PRF 500 and low wall filter and gain was increased until a signal appeared below the cortical bone surface [17].

### Statistical analysis

Qualitative data were expressed as frequencies and proportions. The mean and standard deviation (SD) was calculated for quantitative data. Intra and inter-reader concordance was determined by Cohen’s kappa for qualitative variables. The following cut offs were used for Kappa values: below 0.20 poor, 0.21–0.40 fair, 0.41–0.60 moderate, 0.61–0.80 good, and 0.81–1 excellent [18]. Statistics analysis was using SPSS 15.0 (SPSS, Inc., Chicago, IL, USA).

## Results

Table 1 shows demographic and clinical characteristics of the study participants. The majority was female with 60% being of the polyarticular subtype and a relatively high prevalence of rheumatoid factor at 43%. Low prevalence of synovitis in wrist, second and third MCP was detected clinically. Most of the patients were also on Disease Modifying Antirheumatic Drugs (DMARD) and/or biologic therapies suggesting more severe phenotypes of synovitis in the study.

**Table 1:** Demographic and Clinical characteristics

Age (years) mean ± SD	10.6 ± 5.1
Gender: # female/male	23/7
JIA Subtype n(%)	Polyarticular 18 (60%)
	Oligoarticular 12 (40%)
Disease duration (years) mean ± SD	5.4 + 1.9
Painful joint count mean ± SD	4.9 ± 1.7
Swollen joint count mean ± SD	3.5 ± 1.8
Limited joint count mean ± SD	1.9 ± 0.9
Joints clinically affected n (%)	
Wrist	17 (28)
MCP 2	14 (23)
MCP 3	10 (17)
ESR mm/hr mean ± SD	17.5 ± 14.2
CRP mg/L mean ± SD	15 ± 0.7
Positive Rheumatoid factor number (%)	14 (43%)
Positive Antinuclear antibodies number (%)	19 (57%)
Treatment n (%)	Methotrexate 28 (86%) Sulfasalazine 10 (30%) Biologic therapy 6 (20%) Prednisone 5 (16%)

Table 2 shows the number and percentages of synovitis detected by US in B-Mode and Power Doppler per joint. We observed more synovitis by US than clinical examination as expected (62% vs 28%, 30% vs 23% and 22% vs 17% in the wrist, second and third MCP respectively). The wrist was the most affected joint and MCP 3 the least. Regarding the grade of synovitis we found a higher percentage of grade 1 in the wrist and grade 2 in the MCP joints. Less than 10% of joints evaluated had grade 3 in B-Mode. The PD was present in 60% (18 joints), in one patient the PD was present in bilateral wrist. The grade I was most frequent in all joints and the wrist had more prevalence of PD. Almost all patients had one joint with this abnormal signal.

**Table 2:** Prevalence of synovitis grading in recesses by US grayscale and abnormal PD signal

Joint	Presence of synovitis n (%)	Grayscale grade 1 n (%)	PD grade 1 n (%)	Grayscale grade 2 n (%)	PD grade 2 n (%)	Grayscale grade 3 n (%)	PD grade 3 n (%)
Wrist	37 (62)	15 (30)	6 (10)	17 (28)	0 (0)	5 (8)	2 (3)
MCP 2	18 (30)	5 (8)	3 (5)	8 (13)	1 (2)	5 (8)	3 (5)
MCP 3	13 (22)	2 (3)	3 (5)	8 (13)	0 (0)	3 (5)	0 (0)

The intra-observer concordance for synovitis in all joints was excellent in B-Mode, except for MCP 2, where it was good. For Power Doppler, B-Mode tenosynovitis, cartilage damage and bone erosions it was also excellent. Regarding synovitis grading the concordance was excellent for all grades, except for grade 1 where it was good as observed in Table 3.

**Table 3:** Intra-observer concordance in wrist and MCP joints

Elementary lesions by US	Overall agreement (%) Presence/absence	Kappa Cohen	95% CI	P value
Synovitis				
Wrist	95	.87	.71–1.0	.001
MCP 2	84	.61	.34–89	.001
MCP 3	95	.84	.63–1.0	.001
Abnormal power Doppler signal				
Wrist	95	.87	.71–1.0	.001
MCP 2	100	1.0	1.0–1.0	.001
MCP 3	100	1.0	1.0–1.0	.001
Tenosinovitis flexor tendon				
Grayscale	95	.88	.74–1.0	.001
Power Doppler	100	1.0	1.0–1.0	.001
Cartilage Damage				
MCP 2	94	.82	.59–1.0	.001
MCP 3	97	.94	.84–1.0	.001
Bone erosion	100	1.0	1.0–1.0	.001
Synovitis grading	Agreement (%)	Weighted kappa	95% CI	P value
Synovitis in all joints by US grayscale				
Grade 0	100	1.0	1.0–1.0	.001
Grade 1	73	.61	.43–.83	.004
Grade 2	83	.71	.51–87	.003
Grade 3	87	.84	.63–.91	.001
Abnormal power Doppler signal				
Grade 0	100	1.0	1.0–10	.001
Grade 1	95	.88	.71–1.0	.001
Grade 2	87	.83	.69–.96	.001
Grade 3	100	1.0	1.0–1.0	.001

Table 4 shows the inter-observer reliability. The concordance was excellent for all lesions except for presence of abnormal power Doppler signal in tenosynovitis. Synovitis grading has good concordance for grade 1 and 2 in either B-Mode and Power Doppler. For grade 3 it was excellent.

**Table 4:** Inter-observer concordance in wrist and MCP joints

Elementary lesions by US	Overall agreement (%) Presence/absence	Kappa Cohen	95% CI	P value
Synovitis				
Wrist	96	.90	.80–1.0	.001
MCP 2	92	.81	.67–95	.001
MCP 3	97	.92	.82–1.0	.001
Abnormal power Doppler signal				
Wrist	97	.93	.85–1.0	.001
MCP 2	100	1.0	1.0–1.0	.001
MCP 3	97	.87	.74–1.0	.001
Tenosinovitis flexor tendon				
Grayscale	94	.89	.78–.99	.001
Power Doppler	98	.66	.39–1.0	.001
Cartilage Damage				
MCP 2	94	.89	.78–.99	.001
MCP 3	94	.89	.78–.99	.001
Bone erosion	100	1.0	1.0–1.0	.001
Synovitis grading	Agreement (%)	Weighted kappa	95% CI	P value
Synovitis in all joints by US grayscale				
Grade 0	100	1.0	1.0–1.0	.001
Grade 1	66	.61	.53–.74	.007
Grade 2	69	.64	.57–.86	.004
Grade 3	91	.81	.63–.97	.001
Abnormal power Doppler signal				
Grade 0	100	1.0	1.0–10	.001
Grade 1	67	.63	.58–.91	.004
Grade 2	72	.67	.60–.93	.002
Grade 3	88	.85	.67–.96	.001

The association between joint pain, motion limitation and synovitis detected clinically and synovitis by US was moderate (k = 0.46, CI 95% 0.08–.09).

Although the thickness of the articular cartilage of the MCP was measured and compared with the contralateral joint, it was considered only in a dichotomous manner when there was a decrease in thickness, there was no standard mean, due to the variation in the age of the patients.

Cartilage thinning was seen in 34 (28%) and bone erosion in 12/60 (10%) MCP evaluated respectively, while tenosynovitis, most in flexors tendons, presented in 7/30 (23%) patients,.

## Discussion

To our knowledge, this is the first study that evaluated MSUS reliability for both inflammatory changes and structural damage in wrists and MCPs joints in patients with JIA.

Karmazyn et al. assessed synovitis, tenosynovitis, bone erosions and cartilage thinning in MCP joints by MSUS, finding abnormalities in 32% of 200 joints, however, no data on the reliability of this technique is provided [9]. Other studies have evaluated the concordance of clinical abnormalities and synovitis detected by MSUS and in one study clinical swelling, pain on motion and limitation in the range of motion were significantly associated with MSUS findings at the MCP [8]. Two other studies have shown poor agreement between MSUS and clinical abnormalities [7]. In one study intra-observer reproducibility was excellent, with high kappa values for both B-Mode and PD as in our study [9]. Magni-Manzoni S et al., had observed excellent intra- and inter-reliability for joint effusion, synovial hypertrophy, and PD signal, but had not explored this for specific grades of these lesions [7]. In relation to the synovitis grading, intra and inter-observer concordance was similar to the one observed by the OMERACT-US pediatric task force [10], suggesting that US is a reliable technique. On the other hand, concordance between clinical assessments and US findings was moderate, in line with other studies [7, 8].

### Cartilage damage

One study has demonstrated an acceptable coefficient of variation (16%) between US and MRI for all joints except the wrist, suggesting the US as valid method for measurement of cartilage thickness [19]. The intra and inter-observer concordance relating to cartilage thickness on MCPs were excellent in our sample, similar to reported by Spannow [20]. The importance to detect cartilage loss in children with JIA is due because it represents an early indicator of joint damage and raises the need to intensify therapy before irreversible structural damage development [21].

### Tenosynovitis

In one study, tenosynovitis in wrist was observed in 20% (40) of 200 MCPs evaluated in a longitudinal assessment [9]; this is similar to our study, where we had seen 23% of 30 patients evaluated. Even though, tenosynovitis is commonly seen in the extensor tendons of the wrist [22], we saw more changes in the flexor tendons. Tenosynovitis may be a very relevant finding with some studies in rheumatoid arthritis demonstrating that the presence of tenosynovitis predicts structural damage [23, 24] although it is not known whether the situation in JIA will be similar. The intra observer concordance was excellent for grayscale and PD, while inter-observer concordance was excellent for grayscale and good for PD. As far as we know, there is no study in children in which the reliability of ultrasonography to detect tenosynovitis has been evaluated to compare our results.

### Bone erosions

We evaluated bone erosions only in the MCP joints and not in the wrist, as one study has found wrist changes on MRI, namely carpal depressions, in a large proportion of healthy children [25]. The interpretation of bone irregularities/erosions of the wrist is therefore challenging. [26]. We observed a low prevalence of bone erosions in MCPs in our patients (10%), little less than Karmazyn et al. had found (18% in 36/200 MCPs) [9]. The reliability of the US to detect bone erosions was excellent in our population. However assessing for bony erosive changes in children is difficult because some irregularities in recently ossified bones can be misinterpreted as cortical erosions, highlighting the need for further knowledge of normal bone anatomy throughout the pediatric age groups and for a reference standard like MRI for comparison [27]. Further validation and large-scale studies are required to determine the potential role of US in the detection of bone erosions in children [28].

We consider that including the assessment of tenosynovitis, cartilage damage and bone erosion to the exploration of synovitis in children by ultrasonography, could help the clinician to make a more appropriate therapeutic decision for patients with JIA. Cartilage damage and bone erosions are indicators of joint damage and suggests the need for intensify therapy to prevent major irreversible structural changes [21]. A larger population and long-term follow-up are required to assess the impact this might have.

### Abnormal power Doppler signals

The intra and inter-observer concordance of abnormal power Doppler signals for synovitis was excellent in all regions and good for tenosynovitis. The clear definition of pathologic Doppler signals within an area of synovial hypertrophy [11] helps to improve reliability, given the significant presence of physiologic intraarticular blood flow in children. Doppler signal detection had better reliability than the detection of B-mode changes in other studies [29].

### Limitations

There are some limitations in our study, such as the absence of patients with other disorders like finger pain or hypermobility joint syndrome. In addition, we do not include patients without treatment. Furthermore, we did not use a gold standard examination such as MRI to evaluate the accuracy of the US.

## Conclusions

The intra- and inter-observer reliability of MSUS for inflammatory and structural lesions is good to excellent for the wrist and MCP in patients with JIA.

## Abbreviations

CRP: C-reactive protein
DMARD: Disease Modifying Antirheumatic Drugs
ESR: Erythrosedimentation rate
JIA: Juvenile Idiopathic Arthritis
MCP: Metacarpophallangeal joint
MSUS: Musculoskeletal ultrasound
PD: Power Doppler
SD: Standard deviation
